# Supplementary material for: Identification of photoperiod-induced specific miRNAs in the adrenal glands of Sunite sheep (Ovis aries)
Source: Front Vet Sci. 2022 Jul 22;9:888207. doi: 10.3389/fvets.2022.888207 (PMC9354845; doi:10.3389/fvets.2022.888207)
Supplement: Supplementary file 1 [file Data_Sheet_1.ZIP › Supplementary file/miRDeep2 file.zip!/miRDeep2 file/Know_Structure/LP42A1/oar-mir-758.pdf]

miRBase precursor : oar-mir-758
Total read count : 229
oar-miR-758-5p read count : 0
oar-miR-758-3p read count : 229
remaining reads : 0

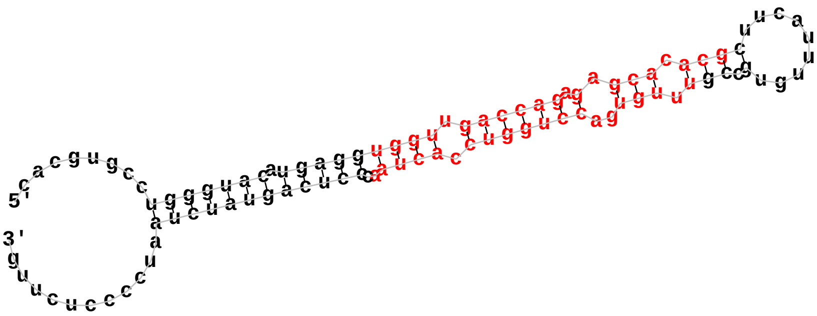

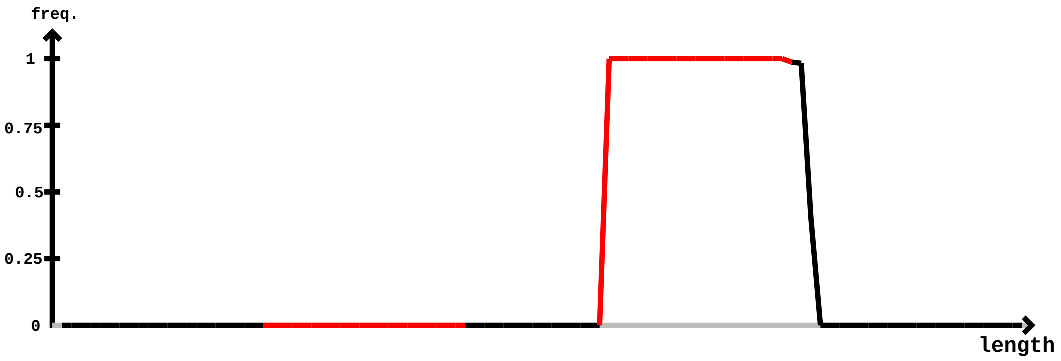


```
                                                        oar-miR-758-3p
                     oar-miR-758-5p
5'- cacgugccuggguacaugaggugguugaccagagagcacacgcuucauuugugccguuugugaccugguccacuaacccucaguaucuaauccccucuug   -3'    exp
    ........((((((.(((((((((.((((((.(.(((.((((.........).))).)))..))))))).)))).)))))))))))...........    reads      mm         sample
    ........................................................uuugugaccugguccacua........................    3          0          seq
    ........................................................uuugugaccugguccacuaa.......................    1          0          seq
    ........................................................uuugugaccugguccacuaac......................    132        0          seq
    ........................................................uuugugaccugguccacuaacc.....................    93         0          seq
```
